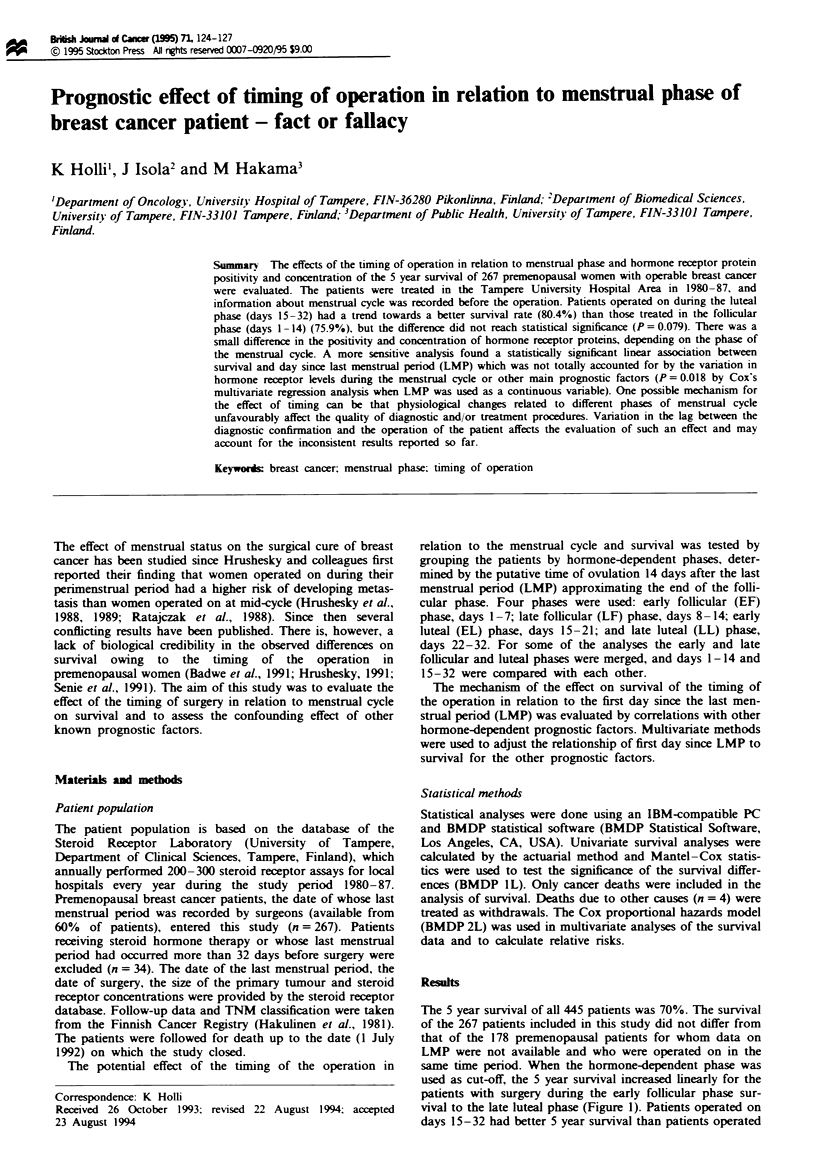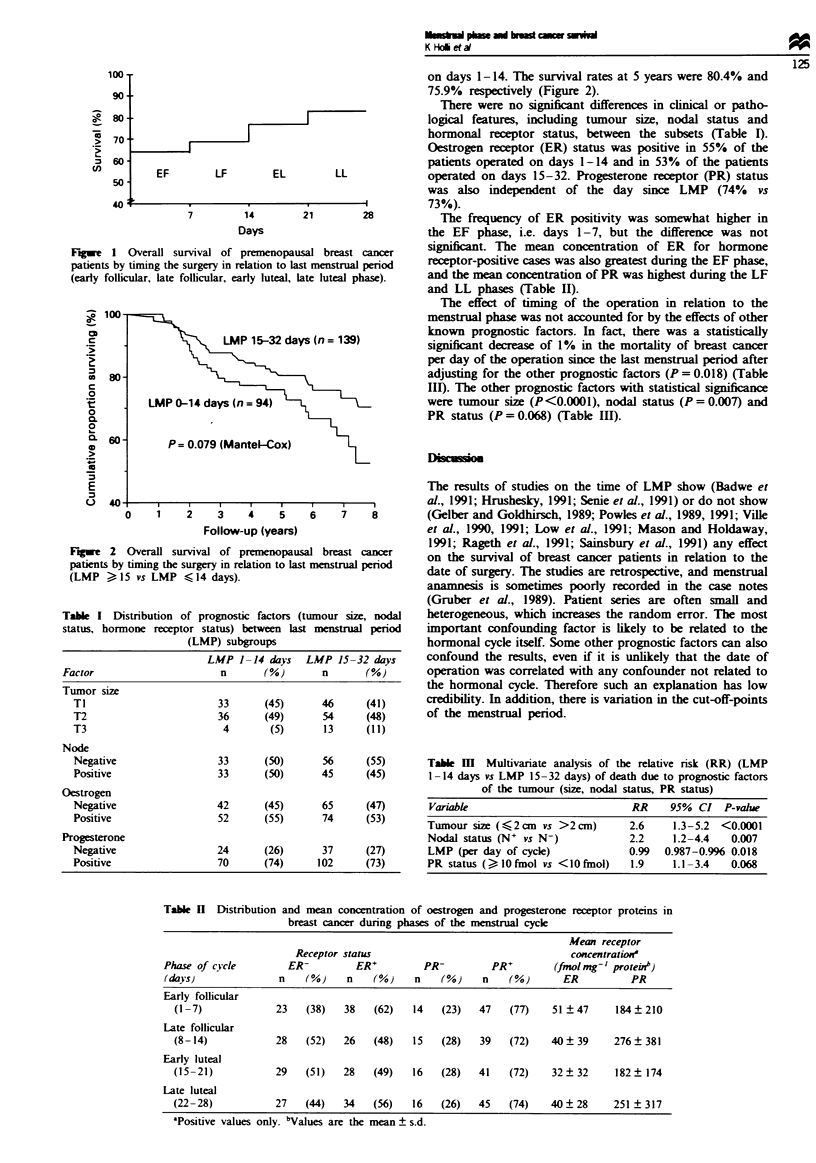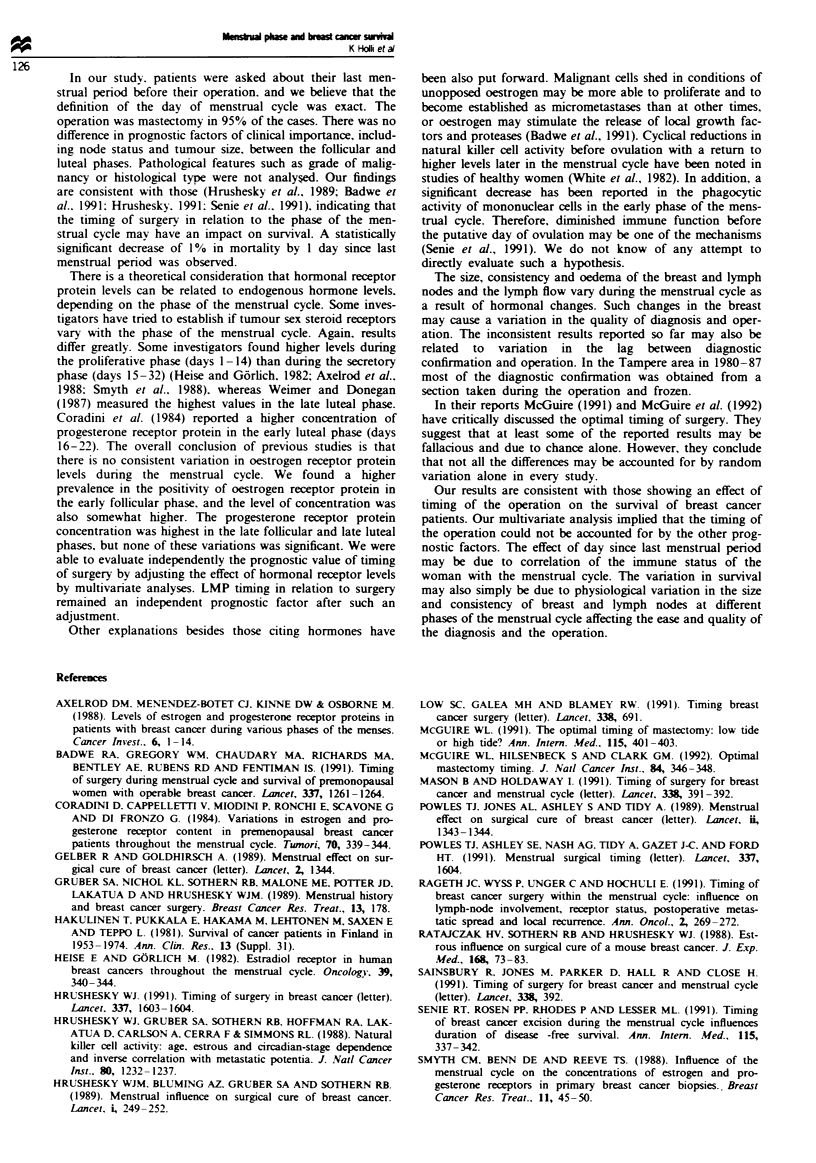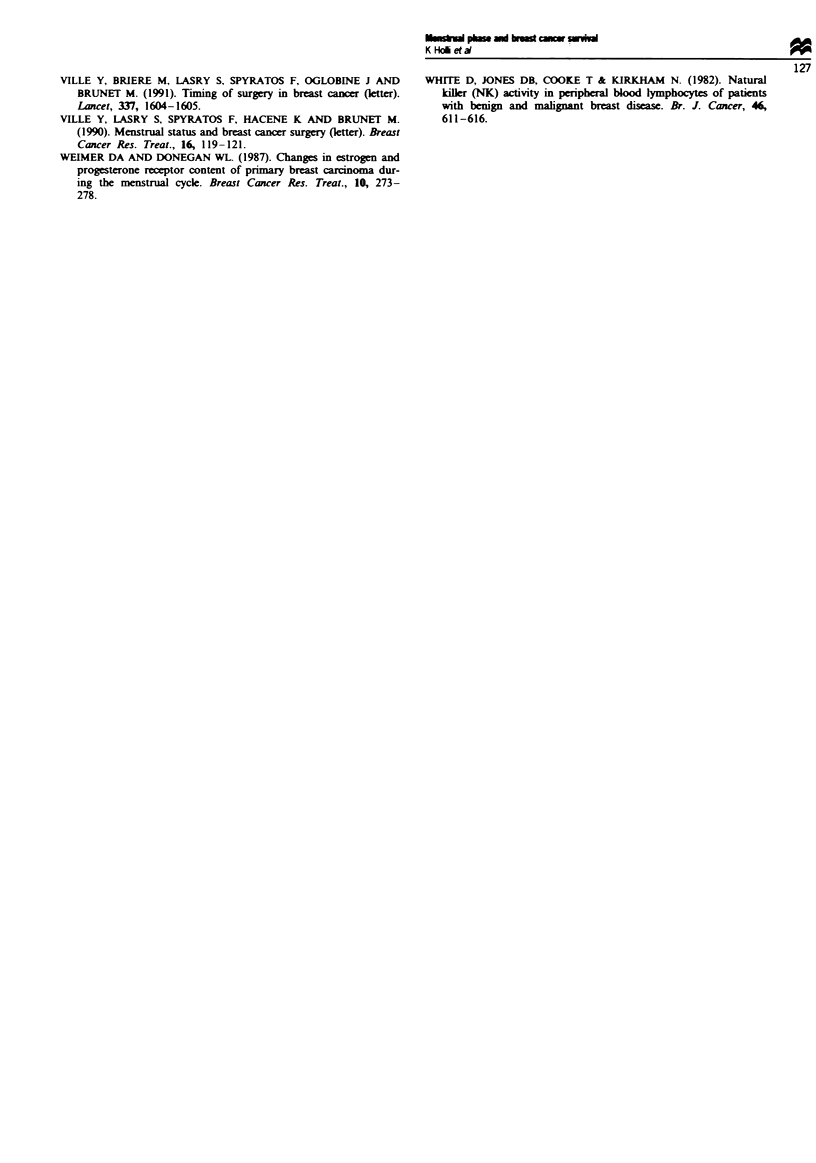# Prognostic effect of timing of operation in relation to menstrual phase of breast cancer patient--fact or fallacy.

**DOI:** 10.1038/bjc.1995.25

**Published:** 1995-01

**Authors:** K. Holli, J. Isola, M. Hakama

**Affiliations:** Department of Oncology, University Hospital of Tampere, Pikonlinna, Finland.

## Abstract

The effects of the timing of operation in relation to menstrual phase and hormone receptor protein positivity and concentration of the 5 year survival of 267 premenopausal women with operable breast cancer were evaluated. The patients were treated in the Tampere University Hospital Area in 1980-87, and information about menstrual cycle was recorded before the operation. Patients operated on during the luteal phase (days 15-32) had a trend towards a better survival rate (80.4%) than those treated in the follicular phase (days 1-14) (75.9%), but the difference did not reach statistical significance (P = 0.079). There was a small difference in the positivity and concentration of hormone receptor proteins, depending on the phase of the menstrual cycle. A more sensitive analysis found a statistically significant linear association between survival and day since last menstrual period (LMP) which was not totally accounted for by the variation in hormone receptor levels during the menstrual cycle or other main prognostic factors (P = 0.018 by Cox's multivariate regression analysis when LMP was used as a continuous variable). One possible mechanism for the effect of timing can be that physiological changes related to different phases of menstrual cycle unfavourably affect the quality of diagnostic and/or treatment procedures. Variation in the lag between the diagnostic confirmation and the operation of the patient affects the evaluation of such an effect and may account for the inconsistent results reported so far.


British Journal of Cancer (1995) 71, 124-127

© 1995 Stockton Press All rights reserved 0007-0920/95 $9.00

### Prognostic effect of timing of operation in relation to menstrual phase of breast cancer patient – fact or fallacy

K Holli[1], J Isola[2] and M Hakama[3]

[1]*Department of Oncology, University Hospital of Tampere, FIN-36280 Pikonlinna, Finland;* [2]*Department of Biomedical Sciences, University of Tampere, FIN-33101 Tampere, Finland;* [3]*Department of Public Health, University of Tampere, FIN-33101 Tampere, Finland.*

**Summary** The effects of the timing of operation in relation to menstrual phase and hormone receptor protein positivity and concentration of the 5 year survival of 267 premenopausal women with operable breast cancer were evaluated. The patients were treated in the Tampere University Hospital Area in 1980-87, and information about menstrual cycle was recorded before the operation. Patients operated on during the luteal phase (days 15-32) had a trend towards a better survival rate (80.4%) than those treated in the follicular phase (days 1-14) (75.9%), but the difference did not reach statistical significance ($P = 0.079$). There was a small difference in the positivity and concentration of hormone receptor proteins, depending on the phase of the menstrual cycle. A more sensitive analysis found a statistically significant linear association between survival and day since last menstrual period (LMP) which was not totally accounted for by the variation in hormone receptor levels during the menstrual cycle or other main prognostic factors ($P = 0.018$ by Cox's multivariate regression analysis when LMP was used as a continuous variable). One possible mechanism for the effect of timing can be that physiological changes related to different phases of menstrual cycle unfavourably affect the quality of diagnostic and/or treatment procedures. Variation in the lag between the diagnostic confirmation and the operation of the patient affects the evaluation of such an effect and may account for the inconsistent results reported so far.

**Keywords:** breast cancer; menstrual phase; timing of operation

The effect of menstrual status on the surgical cure of breast cancer has been studied since Hrushesky and colleagues first reported their finding that women operated on during their perimenstrual period had a higher risk of developing metastasis than women operated on at mid-cycle (Hrushesky *et al.*, 1988, 1989; Ratajczak *et al.*, 1988). Since then several conflicting results have been published. There is, however, a lack of biological credibility in the observed differences on survival owing to the timing of the operation in premenopausal women (Badwe *et al.*, 1991; Hrushesky, 1991; Senie *et al.*, 1991). The aim of this study was to evaluate the effect of the timing of surgery in relation to menstrual cycle on survival and to assess the confounding effect of other known prognostic factors.

### Materials and methods

### *Patient population*

The patient population is based on the database of the Steroid Receptor Laboratory (University of Tampere, Department of Clinical Sciences, Tampere, Finland), which annually performed 200-300 steroid receptor assays for local hospitals every year during the study period 1980-87. Premenopausal breast cancer patients, the date of whose last menstrual period was recorded by surgeons (available from 60% of patients), entered this study ($n = 267$). Patients receiving steroid hormone therapy or whose last menstrual period had occurred more than 32 days before surgery were excluded ($n = 34$). The date of the last menstrual period, the date of surgery, the size of the primary tumour and steroid receptor concentrations were provided by the steroid receptor database. Follow-up data and TNM classification were taken from the Finnish Cancer Registry (Hakulinen *et al.*, 1981). The patients were followed for death up to the date (1 July 1992) on which the study closed.

The potential effect of the timing of the operation in relation to the menstrual cycle and survival was tested by grouping the patients by hormone-dependent phases, determined by the putative time of ovulation 14 days after the last menstrual period (LMP) approximating the end of the follicular phase. Four phases were used: early follicular (EF) phase, days 1-7; late follicular (LF) phase, days 8-14; early luteal (EL) phase, days 15-21; and late luteal (LL) phase, days 22-32. For some of the analyses the early and late follicular and luteal phases were merged, and days 1-14 and 15-32 were compared with each other.

The mechanism of the effect on survival of the timing of the operation in relation to the first day since the last menstrual period (LMP) was evaluated by correlations with other hormone-dependent prognostic factors. Multivariate methods were used to adjust the relationship of first day since LMP to survival for the other prognostic factors.

### *Statistical methods*

Statistical analyses were done using an IBM-compatible PC and BMDP statistical software (BMDP Statistical Software, Los Angeles, CA, USA). Univariate survival analyses were calculated by the actuarial method and Mantel-Cox statistics were used to test the significance of the survival differences (BMDP 1L). Only cancer deaths were included in the analysis of survival. Deaths due to other causes ($n = 4$) were treated as withdrawals. The Cox proportional hazards model (BMDP 2L) was used in multivariate analyses of the survival data and to calculate relative risks.

### Results

The 5 year survival of all 445 patients was 70%. The survival of the 267 patients included in this study did not differ from that of the 178 premenopausal patients for whom data on LMP were not available and who were operated on in the same time period. When the hormone-dependent phase was used as cut-off, the 5 year survival increased linearly for the patients with surgery during the early follicular phase survival to the late luteal phase (Figure 1). Patients operated on days 15-32 had better 5 year survival than patients operated

Correspondence: K Holli
Received 26 October 1993; revised 22 August 1994; accepted 23 August 1994

**Figure 1** Overall survival of premenopausal breast cancer patients by timing the surgery in relation to last menstrual period (early follicular, late follicular, early luteal, late luteal phase).

**Figure 2** Overall survival of premenopausal breast cancer patients by timing the surgery in relation to last menstrual period (LMP ⩾15 *vs* LMP ⩽14 days).

**Table I** Distribution of prognostic factors (tumour size, nodal status, hormone receptor status) between last menstrual period (LMP) subgroups

| *Factor* | *LMP 1–14 days* n | (%) | *LMP 15–32 days* n | (%) |
|---|---|---|---|---|
| Tumor size | | | | |
| T1 | 33 | (45) | 46 | (41) |
| T2 | 36 | (49) | 54 | (48) |
| T3 | 4 | (5) | 13 | (11) |
| Node | | | | |
| Negative | 33 | (50) | 56 | (55) |
| Positive | 33 | (50) | 45 | (45) |
| Oestrogen | | | | |
| Negative | 42 | (45) | 65 | (47) |
| Positive | 52 | (55) | 74 | (53) |
| Progesterone | | | | |
| Negative | 24 | (26) | 37 | (27) |
| Positive | 70 | (74) | 102 | (73) |

on days 1–14. The survival rates at 5 years were 80.4% and 75.9% respectively (Figure 2).

There were no significant differences in clinical or pathological features, including tumour size, nodal status and hormonal receptor status, between the subsets (Table I). Oestrogen receptor (ER) status was positive in 55% of the patients operated on days 1–14 and in 53% of the patients operated on days 15–32. Progesterone receptor (PR) status was also independent of the day since LMP (74% *vs* 73%).

The frequency of ER positivity was somewhat higher in the EF phase, i.e. days 1–7, but the difference was not significant. The mean concentration of ER for hormone receptor-positive cases was also greatest during the EF phase, and the mean concentration of PR was highest during the LF and LL phases (Table II).

The effect of timing of the operation in relation to the menstrual phase was not accounted for by the effects of other known prognostic factors. In fact, there was a statistically significant decrease of 1% in the mortality of breast cancer per day of the operation since the last menstrual period after adjusting for the other prognostic factors ($P = 0.018$) (Table III). The other prognostic factors with statistical significance were tumour size ($P < 0.0001$), nodal status ($P = 0.007$) and PR status ($P = 0.068$) (Table III).

### Discussion

The results of studies on the time of LMP show (Badwe *et al.*, 1991; Hrushesky, 1991; Senie *et al.*, 1991) or do not show (Gelber and Goldhirsch, 1989; Powles *et al.*, 1989, 1991; Ville *et al.*, 1990, 1991; Low *et al.*, 1991; Mason and Holdaway, 1991; Rageth *et al.*, 1991; Sainsbury *et al.*, 1991) any effect on the survival of breast cancer patients in relation to the date of surgery. The studies are retrospective, and menstrual anamnesis is sometimes poorly recorded in the case notes (Gruber *et al.*, 1989). Patient series are often small and heterogeneous, which increases the random error. The most important confounding factor is likely to be related to the hormonal cycle itself. Some other prognostic factors can also confound the results, even if it is unlikely that the date of operation was correlated with any confounder not related to the hormonal cycle. Therefore such an explanation has low credibility. In addition, there is variation in the cut-off-points of the menstrual period.

**Table III** Multivariate analysis of the relative risk (RR) (LMP 1–14 days *vs* LMP 15–32 days) of death due to prognostic factors of the tumour (size, nodal status, PR status)

| *Variable* | *RR* | *95% CI* | *P-value* |
|---|---|---|---|
| Tumour size (⩽2 cm *vs* >2 cm) | 2.6 | 1.3–5.2 | <0.0001 |
| Nodal status ($N^+$ *vs* $N^-$) | 2.2 | 1.2–4.4 | 0.007 |
| LMP (per day of cycle) | 0.99 | 0.987–0.996 | 0.018 |
| PR status (⩾10 fmol *vs* <10 fmol) | 1.9 | 1.1–3.4 | 0.068 |

**Table II** Distribution and mean concentration of oestrogen and progesterone receptor proteins in breast cancer during phases of the menstrual cycle

| *Phase of cycle (days)* | *Receptor status* $ER^-$ n | (%) | $ER^+$ n | (%) | $PR^-$ n | (%) | $PR^+$ n | (%) | *Mean receptor concentration*[a] *(fmol mg*$^{-1}$ *protein*[b]*)* ER | PR |
|---|---|---|---|---|---|---|---|---|---|---|
| Early follicular (1–7) | 23 | (38) | 38 | (62) | 14 | (23) | 47 | (77) | 51 ± 47 | 184 ± 210 |
| Late follicular (8–14) | 28 | (52) | 26 | (48) | 15 | (28) | 39 | (72) | 40 ± 39 | 276 ± 381 |
| Early luteal (15–21) | 29 | (51) | 28 | (49) | 16 | (28) | 41 | (72) | 32 ± 32 | 182 ± 174 |
| Late luteal (22–28) | 27 | (44) | 34 | (56) | 16 | (26) | 45 | (74) | 40 ± 28 | 251 ± 317 |

[a]Positive values only. [b]Values are the mean ± s.d.

In our study, patients were asked about their last menstrual period before their operation, and we believe that the definition of the day of menstrual cycle was exact. The operation was mastectomy in 95% of the cases. There was no difference in prognostic factors of clinical importance, including node status and tumour size, between the follicular and luteal phases. Pathological features such as grade of malignancy or histological type were not analysed. Our findings are consistent with those (Hrushesky *et al.*, 1989; Badwe *et al.*, 1991; Hrushesky, 1991; Senie *et al.*, 1991), indicating that the timing of surgery in relation to the phase of the menstrual cycle may have an impact on survival. A statistically significant decrease of 1% in mortality by 1 day since last menstrual period was observed.

There is a theoretical consideration that hormonal receptor protein levels can be related to endogenous hormone levels, depending on the phase of the menstrual cycle. Some investigators have tried to establish if tumour sex steroid receptors vary with the phase of the menstrual cycle. Again, results differ greatly. Some investigators found higher levels during the proliferative phase (days 1–14) than during the secretory phase (days 15–32) (Heise and Görlich, 1982; Axelrod *et al.*, 1988; Smyth *et al.*, 1988), whereas Weimer and Donegan (1987) measured the highest values in the late luteal phase. Coradini *et al.* (1984) reported a higher concentration of progesterone receptor protein in the early luteal phase (days 16–22). The overall conclusion of previous studies is that there is no consistent variation in oestrogen receptor protein levels during the menstrual cycle. We found a higher prevalence in the positivity of oestrogen receptor protein in the early follicular phase, and the level of concentration was also somewhat higher. The progesterone receptor protein concentration was highest in the late follicular and late luteal phases, but none of these variations was significant. We were able to evaluate independently the prognostic value of timing of surgery by adjusting the effect of hormonal receptor levels by multivariate analyses. LMP timing in relation to surgery remained an independent prognostic factor after such an adjustment.

Other explanations besides those citing hormones have been also put forward. Malignant cells shed in conditions of unopposed oestrogen may be more able to proliferate and to become established as micrometastases than at other times, or oestrogen may stimulate the release of local growth factors and proteases (Badwe *et al.*, 1991). Cyclical reductions in natural killer cell activity before ovulation with a return to higher levels later in the menstrual cycle have been noted in studies of healthy women (White *et al.*, 1982). In addition, a significant decrease has been reported in the phagocytic activity of mononuclear cells in the early phase of the menstrual cycle. Therefore, diminished immune function before the putative day of ovulation may be one of the mechanisms (Senie *et al.*, 1991). We do not know of any attempt to directly evaluate such a hypothesis.

The size, consistency and oedema of the breast and lymph nodes and the lymph flow vary during the menstrual cycle as a result of hormonal changes. Such changes in the breast may cause a variation in the quality of diagnosis and operation. The inconsistent results reported so far may also be related to variation in the lag between diagnostic confirmation and operation. In the Tampere area in 1980–87 most of the diagnostic confirmation was obtained from a section taken during the operation and frozen.

In their reports McGuire (1991) and McGuire *et al.* (1992) have critically discussed the optimal timing of surgery. They suggest that at least some of the reported results may be fallacious and due to chance alone. However, they conclude that not all the differences may be accounted for by random variation alone in every study.

Our results are consistent with those showing an effect of timing of the operation on the survival of breast cancer patients. Our multivariate analysis implied that the timing of the operation could not be accounted for by the other prognostic factors. The effect of day since last menstrual period may be due to correlation of the immune status of the woman with the menstrual cycle. The variation in survival may also simply be due to physiological variation in the size and consistency of breast and lymph nodes at different phases of the menstrual cycle affecting the ease and quality of the diagnosis and the operation.

### References

AXELROD DM, MENENDEZ-BOTET CJ, KINNE DW & OSBORNE M. (1988). Levels of estrogen and progesterone receptor proteins in patients with breast cancer during various phases of the menses. *Cancer Invest.*, **6**, 1–14.

BADWE RA, GREGORY WM, CHAUDARY MA, RICHARDS MA, BENTLEY AE, RUBENS RD AND FENTIMAN IS. (1991). Timing of surgery during menstrual cycle and survival of premonopausal women with operable breast cancer. *Lancet*, **337**, 1261–1264.

CORADINI D, CAPPELLETTI V, MIODINI P, RONCHI E, SCAVONE G AND DI FRONZO G. (1984). Variations in estrogen and progesterone receptor content in premenopausal breast cancer patients throughout the menstrual cycle. *Tumori*, **70**, 339–344.

GELBER R AND GOLDHIRSCH A. (1989). Menstrual effect on surgical cure of breast cancer (letter). *Lancet*, **2**, 1344.

GRUBER SA, NICHOL KL, SOTHERN RB, MALONE ME, POTTER JD, LAKATUA D AND HRUSHESKY WJM. (1989). Menstrual history and breast cancer surgery. *Breast Cancer Res. Treat.*, **13**, 178.

HAKULINEN T, PUKKALA E, HAKAMA M, LEHTONEN M, SAXEN E AND TEPPO L. (1981). Survival of cancer patients in Finland in 1953–1974. *Ann. Clin. Res.*, **13** (Suppl. 31).

HEISE E AND GÖRLICH M. (1982). Estradiol receptor in human breast cancers throughout the menstrual cycle. *Oncology*, **39**, 340–344.

HRUSHESKY WJ. (1991). Timing of surgery in breast cancer (letter). *Lancet*, **337**, 1603–1604.

HRUSHESKY WJ, GRUBER SA, SOTHERN RB, HOFFMAN RA, LAKATUA D, CARLSON A, CERRA F & SIMMONS RL. (1988). Natural killer cell activity: age, estrous and circadian-stage dependence and inverse correlation with metastatic potentia. *J. Natl Cancer Inst.*, **80**, 1232–1237.

HRUSHESKY WJM, BLUMING AZ, GRUBER SA AND SOTHERN RB. (1989). Menstrual influence on surgical cure of breast cancer. *Lancet*, **i**, 249–252.

LOW SC, GALEA MH AND BLAMEY RW. (1991). Timing breast cancer surgery (letter). *Lancet*, **338**, 691.

McGUIRE WL. (1991). The optimal timing of mastectomy: low tide or high tide? *Ann. Intern. Med.*, **115**, 401–403.

McGUIRE WL, HILSENBECK S AND CLARK GM. (1992). Optimal mastectomy timing. *J. Natl Cancer Inst.*, **84**, 346–348.

MASON B AND HOLDAWAY I. (1991). Timing of surgery for breast cancer and menstrual cycle (letter). *Lancet*, **338**, 391–392.

POWLES TJ, JONES AL, ASHLEY S AND TIDY A. (1989). Menstrual effect on surgical cure of breast cancer (letter). *Lancet*, **ii**, 1343–1344.

POWLES TJ, ASHLEY SE, NASH AG, TIDY A, GAZET J-C, AND FORD HT. (1991). Menstrual surgical timing (letter). *Lancet*, **337**, 1604.

RAGETH JC, WYSS P, UNGER C AND HOCHULI E. (1991). Timing of breast cancer surgery within the menstrual cycle: influence on lymph-node involvement, receptor status, postoperative metastatic spread and local recurrence. *Ann. Oncol.*, **2**, 269–272.

RATAJCZAK HV, SOTHERN RB AND HRUSHESKY WJ. (1988). Estrous influence on surgical cure of a mouse breast cancer. *J. Exp. Med.*, **168**, 73–83.

SAINSBURY R, JONES M, PARKER D, HALL R AND CLOSE H. (1991). Timing of surgery for breast cancer and menstrual cycle (letter). *Lancet*, **338**, 392.

SENIE RT, ROSEN PP, RHODES P AND LESSER ML. (1991). Timing of breast cancer excision during the menstrual cycle influences duration of disease -free survival. *Ann. Intern. Med.*, **115**, 337–342.

SMYTH CM, BENN DE AND REEVE TS. (1988). Influence of the menstrual cycle on the concentrations of estrogen and progesterone receptors in primary breast cancer biopsies.. *Breast Cancer Res. Treat.*, **11**, 45–50.

VILLE Y, BRIERE M, LASRY S, SPYRATOS F, OGLOBINE J AND BRUNET M. (1991). Timing of surgery in breast cancer (letter). *Lancet*, **337**, 1604–1605.

VILLE Y, LASRY S, SPYRATOS F, HACENE K AND BRUNET M. (1990). Menstrual status and breast cancer surgery (letter). *Breast Cancer Res. Treat.*, **16**, 119–121.

WEIMER DA AND DONEGAN WL. (1987). Changes in estrogen and progesterone receptor content of primary breast carcinoma during the menstrual cycle. *Breast Cancer Res. Treat.*, **10**, 273–278.

WHITE D, JONES DB, COOKE T & KIRKHAM N. (1982). Natural killer (NK) activity in peripheral blood lymphocytes of patients with benign and malignant breast disease. *Br. J. Cancer*, **46**, 611–616.